# Application of next-generation sequencing technology to study genetic diversity and identify unique SNP markers in bread wheat from Kazakhstan

**DOI:** 10.1186/s12870-014-0258-7

**Published:** 2014-09-28

**Authors:** Yuri Shavrukov, Radoslaw Suchecki, Serik Eliby, Aigul Abugalieva, Serik Kenebayev, Peter Langridge

**Affiliations:** Australian Centre for Plant Functional Genomics, University of Adelaide, Waite Campus, Urrbrae, SA 5064 Australia; Kazakh Research Institute of Agriculture and Crop Production, Almalybak, Kazakhstan

**Keywords:** Bread wheat, Genetic polymorphism, Clusters of unique markers, Genetic phylogeny, Kazakhstan, Next-generation sequencing (NGS), Single nucleotide polymorphism (SNP), Unique markers

## Abstract

**Background:**

New SNP marker platforms offer the opportunity to investigate the relationships between wheat cultivars from different regions and assess the mechanism and processes that have led to adaptation to particular production environments. Wheat breeding has a long history in Kazakhstan and the aim of this study was to explore the relationship between key varieties from Kazakhstan and germplasm from breeding programs for other regions.

**Results:**

The study revealed 5,898 polymorphic markers amongst ten cultivars, of which 2,730 were mapped in the consensus genetic map. Mapped SNP markers were distributed almost equally across the A and B genomes, with between 279 and 484 markers assigned to each chromosome. Marker coverage was approximately 10-fold lower in the D genome. There were 863 SNP markers identified as unique to specific cultivars, and clusters of these markers (regions containing more than three closely mapped unique SNPs) showed specific patterns on the consensus genetic map for each cultivar. Significant intra-varietal genetic polymorphism was identified in three cultivars (Tzelinnaya 3C, Kazakhstanskaya rannespelaya and Kazakhstanskaya 15). Phylogenetic analysis based on inter-varietal polymorphism showed that the very old cultivar Erythrospermum 841 was the most genetically distinct from the other nine cultivars from Kazakhstan, falling in a clade together with the American cultivar Sonora and genotypes from Central and South Asia. The modern cultivar Kazakhstanskaya 19 also fell into a separate clade, together with the American cultivar Thatcher. The remaining eight cultivars shared a single sub-clade but were categorised into four clusters.

**Conclusion:**

The accumulated data for SNP marker polymorphisms amongst bread wheat genotypes from Kazakhstan may be used for studying genetic diversity in bread wheat, with potential application for marker-assisted selection and the preparation of a set of genotype-specific markers.

**Electronic supplementary material:**

The online version of this article (doi:10.1186/s12870-014-0258-7) contains supplementary material, which is available to authorized users.

## Background

Genotyping of cultivars and breeding material using molecular markers is a very important tool for modern plant breeders. As a component of marker-assisted selection (MAS), molecular markers improve and accelerate the process of development and release of new cultivars [1-4]. Molecular markers also facilitate unequivocal cultivar identification and resolution of potential ownership conflicts by elucidating the pedigree/ancestry of a given genotype. They are also used to investigate genetic distance and similarity within germplasm collections [5].

Single nucleotide polymorphism (SNP) markers are one of the most important types of molecular markers and there have been significant recent developments in this technology for cultivated bread wheat, *Triticum aestivum* L. [6-9]. The initial development of SNP markers was cumbersome and low-throughput, utilising EST [10-13] and BAC sequencing information [14]. However, whole genome profiling [15] through Next-generation sequencing (NGS) platforms has greatly facilitated the development and deployment of SNP platforms for plant breeding [2,16], particularly for crops such as wheat [3,17,18]. The development of NGS technologies and associated data management tools for genotyping of allo-polyploid species [19] has been critical for bread wheat, a species with a very large genome and three sets of homoeologous chromosomes: AA, BB and DD [20-24]. Three general types of NGS-SNP technologies for wheat genotyping have been exploited [9]. The simplex technology is commonly deployed with a TaqMan assay for genotyping a single SNP amongst multiple samples [25]. Multiplex genotyping methods are suitable for studying a larger number of SNP markers (30–60) across a limited numbers of samples, typically fewer than several hundred [13,26]. In our study we selected a third, array-based technology that can assay thousands of SNP markers across the genome. We used an Infinium 9 K SNP array [3,22,27] to investigate the genetic diversity amongst a small panel of bread wheats from Kazakhstan to access the relationship between Kazakh wheats and elite lines from other regions. Similar strategies for SNP genotyping have also been used for durum wheat [28-30], emmer wheat [31], synthetic wheat and their wild progenitors [32,33].

SNP genotypes have been useful for both linkage and association mapping approaches to associate markers with agronomic traits. Using KASPar technology, high density SNP genetic maps have been established for doubled-haploid populations [24] and for sorted chromosome 3B [34]. Genetic relationships were studied in 172 European winter bread wheat lines, although only 518 of 1,395 high quality SNP markers were found to be suitable for use in association mapping [35].

Further modifications of NGS-SNP technology were developed and applied to the study of genetic diversity in durum wheat. Single strand conformation polymorphism (SSCP) and KASPar technology were exploited for fine mapping ‘Gene of Interest’ in durum wheat lines with bulked segregant analysis [36]. Sequence related amplified polymorphism (SRAP) markers and Genotyping-by-Sequencing (GBS) technologies were successfully used for genotyping wheat germplasm, cultivars and mapping populations [37-39]. In diploid species *Aegilops tauschii*, the progenitor of the wheat D genome, 7 K - 185 K polymorphic and high-confidence SNP markers were reported and used for studying of the genome evolution, genetic diversity and geographic origin of various accessions [33,40-42]. Bioinformatics databases including CerealsDB and SNPMeta provide publicly accessible data on NGS-SNP, GBS and KASPar technology [43,44].

The aims of the current study were: (1) assess the suitability of the SNP platforms to explore the origins and basis of selection for relatively isolated wheat improvement programs using Kazakhstan as a model; (2) identify polymorphic and unique SNP markers and examine their distribution across the genome and chromosomes of a set of ten bread wheat cultivars from Kazakhstan, including eight cultivars recently bred and two historical reference-pedigree cultivars; and (3) assess intra- and inter-varietal genetic diversity among the selected cultivars to explore the origins of key Kazakh wheat lines. This initial study can be used as a basis for further a more extensive germplasm study and to develop a set of SNP markers useful for breeding programs in Kazakhstan.

## Methods

### Plant material

Seeds of ten cultivars of bread wheat were received from the Kazakh Research Institute of Agriculture and Crop Production, Almalybak, Kazakhstan, and are listed with their pedigree description in Table 1. Agro-ecological conditions in Kazakhstan, where the cultivars are grown, can be characterised as strong drought during the entire vegetation period with localised salinity stress.

**Table 1:** **Bread wheat germplasm used for 9 K Infinium array SNP marker development**

**Code**	**Name**	**Year of release**	**Pedigree**
AKT-39	Aktobe 39	2007	Bezostaya 1/Orenburgskaya 1
AST-2	Astana 2	2008	VIR 264–2/Tselinnaya yubileinaya/BC3
*ERY-841*	*Erythrospermum 841*	*1929*	*Ashkhabad (Locals)*
KAR-90	Karabalykskaya 90	1994	Lutescence 5714/Tselinnaya 21
KAZ-R	Kazakhstanskaya rannespelaya	1990	Novosibirskaya 67/Omskaya 9
KAZ-15	Kazakhstanskaya 15	1992	Priboi/Lutescence 49-71-62
KAZ-19	Kazakhstanskaya 19	1994	Chaika/Saratovskaya 29
PAV-93	Pavlodarskaya 93	1999	Mironovskaya 808/Krasnaya Zvezda/K-4115
*SAR-29*	*Saratovskaya 29*	*1957*	*Albidum 24/Lutescens 55-11*
TZE-3C	Tzelinnaya 3C	1996	Shortandinskaya 25/Lee/Kenya/Lutescence 104-6-24

Eight cultivars are derived from recent breeding programs in Kazakhstan along with two historical reference-pedigree cultivars (shown in Italics). Pedigree information is extracted from publications and databases [37,45-47].

### Selection of wheat accessions with published data for phylogenetic comparison

A small panel of wheats of wide geographic origin was selected from 2,994 wheat accessions with published data [3] for their comparison with studied cultivars from Kazakhstan. Genetically diverse accessions were selected from: Asia (4), Australia (9), Canada (8), China (6) and USA (10) (Additional file 1). All accessions were spring wheat cultivars, with the exception of two landraces from Uzbekistan. The panel was used for phylogenetic comparison with the ten cultivars from Kazakhstan used in this study.

### DNA extraction and 9 K Infinium SNP marker analysis

Plants were grown in a greenhouse until tillering and five uniform plants were selected for each cultivar, with the exception of cultivar TZE-3C, where six plants were chosen. DNA was extracted from a single young leaf from each plant, using a phenol-chloroform extraction method [48]. DNA concentration was adjusted to 50 ng μl^−1^ and the quality of DNA was checked both by gel electrophoresis and with PCR. DNA samples (51 in total) were submitted to the Department of Primary Industries (DPI), Victoria (Australia) for genotyping using the 9 K Infinium SNP array [3,18,22,27] with details presented in Additional file 2.

All SNP markers were assessed and polymorphic markers were identified where alleles differed in at least one cultivar. Unique SNP markers were distinguished from the polymorphic ones, defined as being diagnostic for a single cultivar (Singleton). Additionally, clusters of three or more unique markers, which mapped to small regions of a single chromosome on the consensus map (2.5 cM or less between neighbouring SNPs), were defined and identified for each cultivar. Unique SNP markers with identical locations on the genetic map were counted as a single marker when identifying SNP marker clusters. All unique SNP markers and their clusters were assessed and checked manually. Markers, which were polymorphic among the 5–6 plants within a given cultivar were classified as ‘intra-varietal’, and not used for ‘inter-varietal’ analysis.

### Computer map imaging and molecular phylogeny

The computer software program FlapJack [49] was used for graphical genotyping and visualization of the genetic map for all samples and to indicate the positions of SNP markers with known chromosome locations. A similarity matrix exported from FlapJack and from Table of SNP markers [3] was converted into a distance matrix, which was then applied to construct a BioNJ trees [50] using SplitsTree4 program [51,52]. Nei’s gene diversity and Polymorphism Information Content (PIC) were calculated using PowerMarker, version 3.25 [53]. Nei’s gene diversity determines the probability that two randomly selected alleles are different among samples. PIC indicates the probability that two randomly selected samples will show polymorphic alleles. MapChart, version 2.2, was used for the preparation and visualization of a genetic map [54].

## Results

### Distribution of polymorphic SNP markers on wheat genomes and chromosome groups

The 9 K Infinium SNP analysis identified 8,632 SNP markers, of which 5,898 were effective across the ten Kazakh wheat cultivars. The data set supporting the results of this article is included in Additional file 3. Nei’s gene diversity was very high but relatively similar between the cultivars, ranging from 0.5044 (PAV-93) to 0.5309 (*ERY-841*), and PIC values ranged between 0.3832 and 0.4224.

However, only 2,730 of the polymorphic SNP markers (46.3%) could be mapped to a chromosome location in wheat and used for further study (Table 2). These markers were distributed almost equally across the A and B genomes (1,341 and 1,242 markers, respectively), while approximately 10-fold fewer SNP markers were mapped in the D genome. The distribution of SNP markers across homeologous chromosome was not highly variable and ranged between 279 (group 4) and 484 (group 2) SNP markers.

**Table 2:** **The distribution of polymorphic SNP markers across the A, B and D genomes of hexaploid wheat**

**Chromosome**	**Genome**			**Total**
	**A**	**B**	**D**	
1	233	141	45	419
2	163	295	26	484
3	154	136	7	297
4	200	70	9	279
5	194	244	34	472
6	175	242	18	435
7	222	114	8	344
Total	1341	1242	147	2730

A total of 2,730 markers were found to be polymorphic across ten cultivars of bread wheat from Kazakhstan.

### Distribution of unique SNP markers mapping to individual chromosomes

Unique markers were defined as those diagnostic for a single cultivar of the ten included in this study. There were 885 unique SNP markers identified, accounting for a high proportion (32.4%) of the total number of mapped markers (Table 3).

**Table 3:** **Distribution of 885 unique SNP markers across chromosomes for each of ten bread wheat cultivars from Kazakhstan**

**Chromosome**		**Cultivar**										**Total**
		**PAV-93**	**KAR-90**	**TZE-3C**	***SAR-29***	**AST-2**	**KAZ-R**	**AKT-39**	**KAZ-15**	**KAZ-19**	***ERY-841***	
**1**	**A**			3	3				11	15	17	49
	**B**				2			10		11	21	44
	**D**									3	3	6
**2**	**A**	1			10	1				2	75	89
	**B**		1		1	2			3	12	63	82
	**D**					1			1	3	3	8
**3**	**A**	4	2					2		8	9	25
	**B**		7				5	1	3	7	28	51
	**D**							2		1		3
**4**	**A**	1		22		4		5		1	7	40
	**B**				1	17				10	16	44
	**D**								1	1		2
**5**	**A**		2		1	4	2	16	1	4	22	52
	**B**		2		1		3	2	1	25	82	116
	**D**							13		4	3	20
**6**	**A**		6			1		9	2	2	37	57
	**B**		11		1			17	4	9	6	48
	**D**						1			2	1	4
**7**	**A**				1			3		33	50	87
	**B**							4		17	29	50
	**D**	1						3	1		3	8
**Total**		7	31	25	21	30	11	87	28	170	475	885

The distribution of the unique SNP markers was disproportional between the studied cultivars. More than half (53.6%) were specific to the old, reference-pedigree cultivar *ERY-841*, indicating that this genotype was the most genetically distinct from the other wheat cultivars. The second and third most genetically distinctive cultivars were KAZ-19 and AKT-39, possessing 19.2% and 9.8%, respectively, of the total number of identified unique SNP markers. The least genetic polymorphism was found in two cultivars, KAZ-R and PAV-93, associated with only 1.2% and 0.8% of the total number of unique SNP markers, respectively (Table 3).

### Distribution of clusters of unique SNP markers across chromosomes

**Figure 1:**
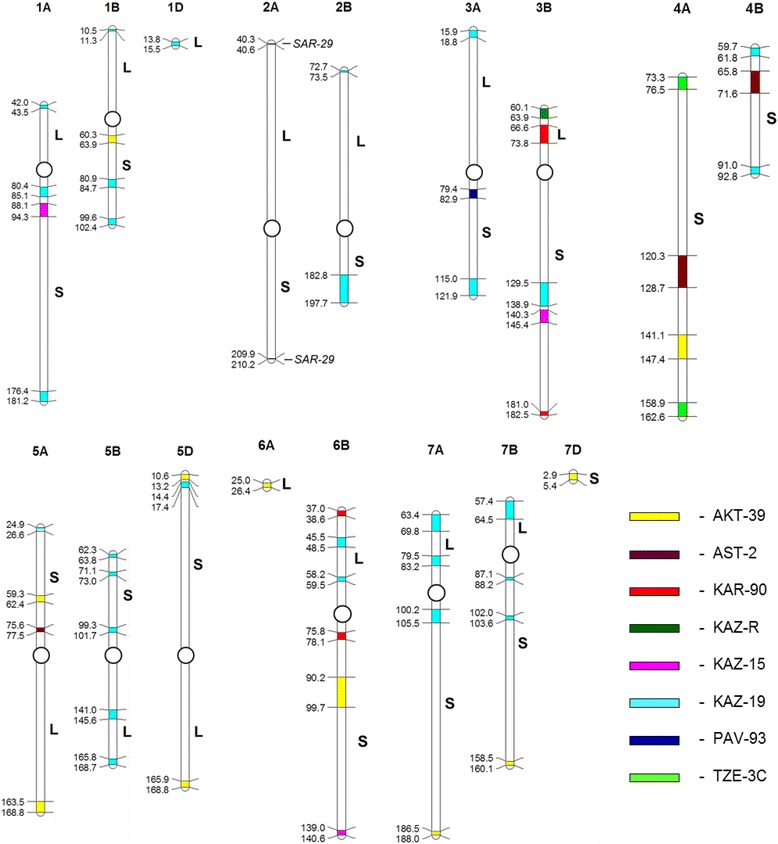
**Distribution of clusters of unique SNP markers (regions containing three or more closely mapped SNPs) by colour code on the consensus genetic map.** Coloured segments of the chromosomes for eight cultivars from Kazakhstan are coded as indicated in the key on the right of the image. The reference-pedigree cultivar *ERY-841* is not shown because of the extensive diversity present in this genotype. Clusters found for *SAR-29* are shown directly on chromosome 2A. Genetic distances are presented in cM on the left of each chromosome; chromosomes are oriented as they are described in the consensus map, and show long (L) and short (S) arms. Approximate positions of centromeres are indicated by open circles. The map was constructed using MapChart software [54], where initial data for clusters of unique SNP markers were transferred from matrix data generated by DPI, Victoria (Australia).

The distribution of the unique SNP markers mapped to individual chromosomes was specific for each cultivar. All chromosomes were covered by unique SNPs but their distribution frequencies were different. In contrast to individual unique SNP markers, clusters of three or more closely mapped markers were identified for each cultivar, and their distribution patterns were more specific and could be used to characterize the studied germplasm (Figure 1). Clusters of unique SNP markers may represent segments of the wheat chromosomes introgressed from exotic ancestral germplasm. Such clusters of unique SNP markers are diagnostic due to their presence in only one cultivar. Almost all groups of three or more SNP markers listed in Table 3 were mapped to small chromosomal regions and could thus be defined as clusters, with the exception of only a few SNPs, dispersed along the chromosomes in different cultivars. The most diverse cultivar *ERY-841*, with the highest number of unique SNP markers, also had 35 clusters of unique SNPs covering almost all chromosomes. *ERY-841* is not shown in Figure 1 because of the extensive diversity found in this cultivar. The next two most diverse cultivars, in terms of unique SNPs (KAZ-19 and AKT-39), had 29 and 11 clusters of unique SNP markers, respectively, spread across genomes and chromosomes (Figure 1). Importantly, clusters of unique SNP markers were distributed very specifically for each of the studied genotypes. For example, single clusters of unique SNP markers were found in PAV-93 and KAZ-R on chromosomes 3AS and 3BL, respectively, while two clusters were found in TZE-3C, both located on chromosome 4AS. The density of unique SNP markers within each cluster also varied. For example, four SNP markers spaced at approximately 2 cM intervals formed a cluster on chromosome 4A in AST-2 (120.3-128.7 cM), while a similar sized region (65.8-71.6 cM) on chromosome 4B contained 17 SNP markers in the same cultivar. Similarly, in AKT-39, the densities of SNP markers in each of the 11 clusters along nine chromosomes were variable. Interestingly, the two clusters of 10 unique SNP markers in *SAR-29* were particularly small, amounting to 0.3 cM fragments each on the very distal parts of chromosome 2A (this could not be shown by colour-coding on the genetic map in Figure 1). No clusters of unique SNP markers were found for some chromosomes of the D genome, whilst only a single cluster was identifiable on each of chromosomes 6AL, 1DL and 7DS. The most diverse chromosomes in this study were 3B and 6B, each with clusters of unique SNPs for four cultivars (Figure 1).

### Genotyping and intra-varietal polymorphism

**Figure 2:**
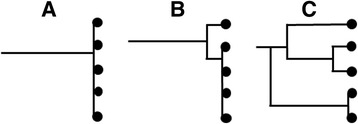
**Three types of intra-varietal polymorphism depicted using mini-dendrogram cartoons, as identified by SNP marker analysis of ten cultivars of bread wheat from Kazakhstan.** Dots represent plants from a single cultivar and lines indicate differences/similarity between them. **(A)** No or very low intra-varietal genetic polymorphism; **(B)** Moderate intra-varietal genetic differences for one plant; **(C)** Significant intra-varietal polymorphism with diverse genotypes.

Five to six independent DNA samples from individual plants were used to assess intra-varietal genetic polymorphism for each cultivar by SNP marker analysis (Figure 2). The ten cultivars were categorised into three groups. Five genotypes (AKT-39, *ERY-841*, KAZ-19, *SAR-29* and PAV-93) had no or very low intra-varietal polymorphism, and plants of these cultivars can be described as genetically uniform (Figure 2A). The second group is represented by two cultivars (AST-2 and KAR-90) with moderate intra-cultivar polymorphism, where one of five studied individuals showed different SNP marker alleles in a few genetic regions across different chromosomes (Figure 2B). This polymorphism may represent residual heterozygocity in the cultivars, which is still segregating. Three cultivars (KAZ-R, KAZ-15 and TZE-3C) fall into a third group and showed significant genetic intra-varietal polymorphism, showing that they represent mixtures of genetically divergent individuals (Figure 2C). SNP marker analysis indicated that the genetic diversity was smaller in two of these cultivars (KAZ-R and KAZ-15) but much wider in TZE-3C, where genetic variability among two groups of individuals within this cultivar was comparable with inter-varietal polymorphism. We suggest that high levels of intra-varietal genetic polymorphism in the third group may reflect the method employed for breeding these cultivars (e.g. population rather than linear methods of selection).

**Figure 3:**
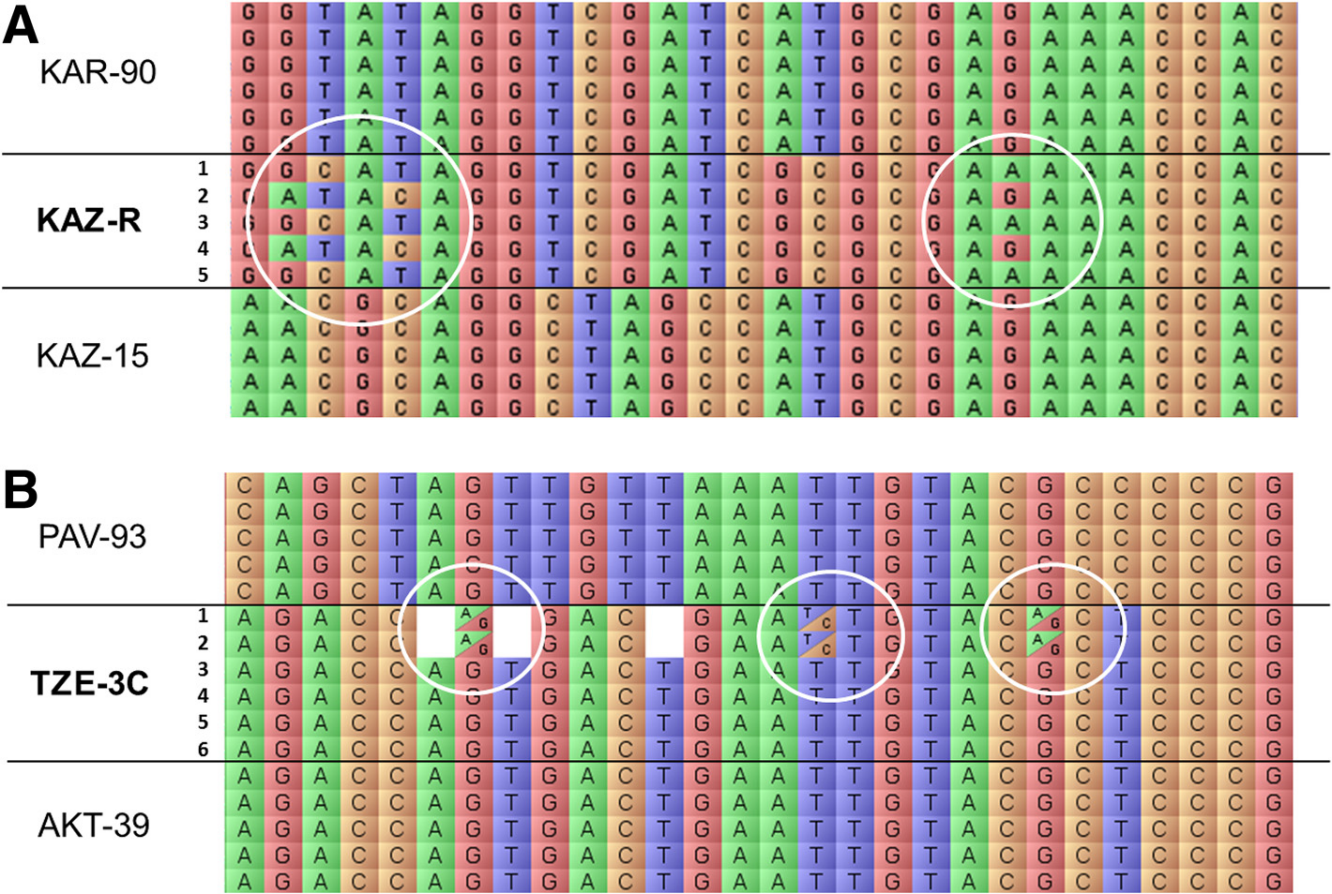
**Examples of intra-varietal polymorphism seen in bread wheat cultivars, as visualised using FlapJack software [** 49 **].** Columns show nucleotides in coloured boxes at the SNP positions for each marker, and each line characterises the genotyping of an individual plant. Cultivar names are given at the left of the image, where cultivars with intra-varietal polymorphism are indicated in Bold. **(A)** Intra-varietal polymorphisms on chromosome 3B among five plants in cultivar KAZ-R are indicated by white circles. **(B)** Heterozygous SNP markers on chromosome 4A among 6 plants in cultivar TZE-3C are shown as boxes with a diagonal line within white circles. No score results for several SNPs are shown as empty boxes.

An example of the intra-varietal polymorphism in cultivar KAZ-R is presented in Figure 3A. The genotyping of five individuals in this cultivar can clearly distinguish the mix of two groups of genetically diverse plants: (i) № 1, 3 and 5, and (ii) № 2 and 4. Heterozygous SNP markers were sometimes registered in the three cultivars showing significant intra-varietal polymorphism, with the majority of then in cultivar TZE-3C (Figure 3B). However, their frequencies did not exceed 1% of mapped polymorphic SNPs.

### Inter-varietal polymorphism and molecular-genetic phylogeny

**Figure 4:**
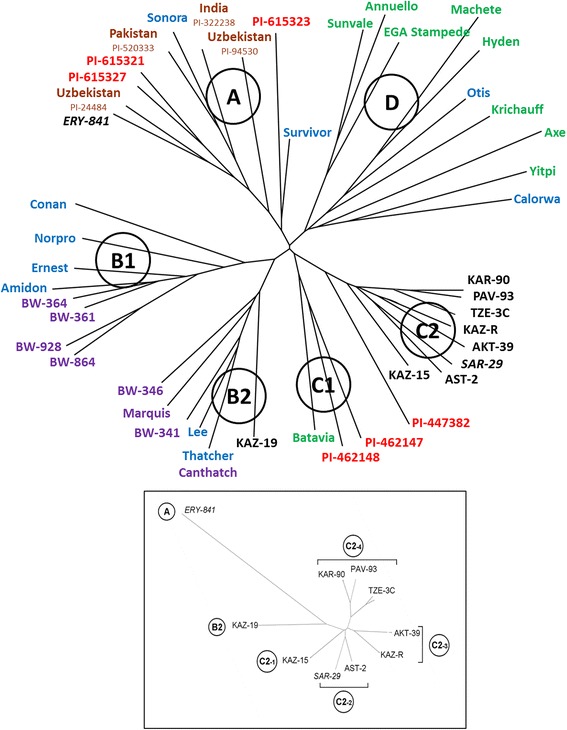
**Molecular-genetic phylogeny of ten cultivars of bread wheat from Kazakhstan among a small germplasm diversity panel.** The panel of international wheat genotypes and ten studied cultivars from Kazakhstan are shown with circled numbers for phylogenetic clades and sub-clades in the top part of the figure. Geographic origins of the reference wheat genotypes are colour-coded: red (China), brown (other Asian countries), blue (USA), purple (Canada), green (Australia), and black (Kazakhstan). Extracted results for ten cultivars from Kazakhstan are presented separately in a framed insert in the bottom part of the figure, where clade, sub-clades and clusters are shown in circles. Eight recent cultivars bred in Kazakhstan are shown in normal font while two older, reference-pedigree cultivars are shown in Italics. Data are shown by BioNJ trees based on 5,898 polymorphic SNP markers in the present study and extracted from published data [3].

Despite a common geographic origin for all of the studied wheat cultivars, SNP marker analysis demonstrated that they have broad inter-varietal genetic polymorphism (Figure 4). As expected, the most genetically distant genotype was the reference-pedigree cultivar *ERY-841*, the sole member of clade A. Cultivar KAZ-19 was most closely related to *ERY-841*, but was also genetically distinct from all other cultivars in this study (sub-clade B2). The remaining eight genotypes were categorised into four clusters of a single sub-clade C2. While the cultivar KAZ-15 (cluster C2-1) was developed at the same breeding centre as KAZ-19, they are genetically divergent. Two cultivars (AST-2 and the reference-pedigree cultivar *SAR-29*) fell into cluster C2-2, while two cultivars (AKT-39 and KAZ-R) were classified in cluster C2-3. The three remaining cultivars (TZE-3C, PAV-93 and KAR-90) fell into cluster C2-4, where PAV-93 and KAR-90 were closely related (Figure 4).

The genetic polymorphism analysis of the ten studied lines was compared with a panel of representative diverse cultivars of spring bread wheats of broad geographical origin extracted from published data [3] (Figure 4). Reference-pedigree cultivar *ERY-841* was most distinct, sharing clade A with the very old American cultivar Sonora, together with genotypes from China and South-Central Asia. Both parts of clade B represented genotypes from North America (USA and Canada), but also included the modern Kazakh cultivar KAZ-19 (sub-clade B2). Clade C was quite diverse, with all remaining cultivars from Kazakhstan falling into a single sub-clade C2. Genetic differences between clusters in sub-clade C2 were significant but much less when compared to genotypes in other clades. Only cultivars from Australia and the USA were found in clade D, indicating minimal genetic relationships with the genotypes from Kazakhstan (Figure 4).

## Discussion

SNP markers are a powerful tool for the study of genetic polymorphism, molecular phylogeny and for MAS [6-9]. Ten cultivars of bread wheat from Kazakhstan were analysed with 8,632 SNP markers for intra- and inter-varietal genetic polymorphism. More than half of the markers (68.3%) were polymorphic, and these were used for further molecular-genetic study. The proportion of polymorphic SNP markers was similar to those previously published for both small and large sets of bread wheat germplasm: 20 US wheat cultivars and 13 diverse genotypes [12], and 478 spring and winter wheat lines [21], respectively. Using the same 9 K Infinium SNP marker array for 172 elite bread wheat lines, Würschum et al. [35] reported that only 16.2% of SNP markers were polymorphic across the collection. However, only 1,395 identified SNPs were ‘high-quality’ markers, because a relatively large portion of SNP markers with either missing values or high heterozygosity [35]. Recent results with a 90 K SNP array for 550 hexaploid and 55 tetraploid accessions of wheat identified 15.4% and 25.9% of the total number of SNP markers as being polymorphic for Australian and European material, respectively [18]. These results were similar to an earlier report [23], where 19.5% of 500 K SNPs were identified as polymorphic among eight UK bread wheat cultivars. The proportion of useful polymorphic markers obtained from NGS technologies depends largely on the data quality. It has been reported that the proportion of polymorphic SNP markers can be increased with deeper sequence coverage. For example, an increase from 66% to 83% of total SNP markers in a collection of durum wheats was achieved, when sequence coverage was increased from 12-fold to 16-fold [36]. However, the cost increases associated with enhanced sequence coverage may not justify a relatively moderate increase in polymorphic SNPs.

In the present study, 2,730 (46.3%) of the polymorphic SNP markers could be mapped to a chromosomal location, higher than the proportions reported in similar studies, of 37.1% [35] and 22.5% [18] and the distribution of the mapped polymorphic SNP markers across the A, B and D genomes was similar to previous reports for both SNP markers [12,18,27,32,35,38] and SSR markers [55]. Polymorphic SNP markers were distributed more or equally between the A and B genomes, while there were fewer markers mapping to the D genome. The D genome in hexaploid wheat is the youngest one of the three, thought to have been introduced to hexaploid wheat around 8,000 years ago as a result of natural hybridisation with *Aegilops tauschii* (DD) [56]. By comparison, hybridisation between progenitors of the A and B genomes is estimated to have occurred between 0.58-0.82 million years ago, which is significantly older than suggested earlier [57]. The relatively recent introgression and narrow genetic diversity of the D genome may have led to the low SNP rate seen in our and others’ observations, since genetic diversity is likely dependent on stochastic processes during wheat evolution [32].

There were no strong trends noted for the frequencies of SNP markers in each of the different chromosomes. Table 4 lists chromosomes with the highest and lowest frequencies of mapped SNP markers in genomes of hexaploid and tetraploid wheats. Reasons for the observed variability may be related to the different sets of SNP markers developed, and the broad diversity in wheat genotypes used across the different studies. Interestingly, there was a tendency in the studies of bread wheat for low frequencies of SNPs on chromosomes 4A, 4B, 7B, 3D and 4D (Table 4). A low frequency of molecular markers located on chromosome 4A of bread wheat may be related to ancient, non-homeologous translocations having occurred in this chromosome [32,58,59].

**Table 4:** **Chromosomes with the highest and lowest frequencies of polymorphic SNP markers on chromosomes in hexaploid wheat reported in recent studies**

**Highest frequency**		**Lowest frequency**		**Total polymorphic SNP markers used in each genome**	**Species**	**Reference**
**Chromosome**	**%**	**Chromosome**	**%**			
**A genome**						
1A	17.4	3A	11.5	1341	Bread wheat	Present study
1A	18.9	4A	11.6	397	Bread wheat	[27]
2A	26.5	4A	3.8	370	Bread wheat	[24]
6A	20.6	4A	10.8	660	Bread wheat	[24]
7A	20.5	4A	6.8	146	Bread wheat	[12]
5A/7A	18.9	1A	9.8	143	Bread wheat	[35]
7A	17.2	3A	11.9	16662	Bread wheat	[18]
6A	20.0	5A	8.7	310	Durum wheat	[29]
7A	17.1	5A	11.4	516	Durum wheat	[30]
7A	16.5	5A	10.8	613	Wild emmer	[31]
**B genome**						
2B	23.8	4B	5.6	1242	Bread wheat	Present study
2B	31.5	4B	4.2	289	Bread wheat	[24]
2B	27.6	4B	6.5	23411	Bread wheat	[18]
1B	23.9	7B	7.1	896	Bread wheat	[24]
5B	22.7	4B	5.8	445	Bread wheat	[27]
5B	20.4	4B/7B	12.0	142	Bread wheat	[35]
3B	17.9	6B	10.4	134	Bread wheat	[12]
7B	20.9	5B	6.0	398	Durum wheat	[29]
6B	19.3	4B	10.7	430	Durum wheat	[30]
6B	18.3	3B	11.2	492	Wild emmer	[31]
**D genome**						
1D	30.6	3D	4.8	147	Bread wheat	Present study
5D	23.7	3D	5.1	59	Bread wheat	[27]
6D	29.8	3D	2.1	94	Bread wheat	[24]
2D	26.6	4D	3.4	237	Bread wheat	[24]
2D	22.6	4D	4.6	6904	Bread wheat	[18]
2D/3D	22.2	4D/5D	5.6	18	Bread wheat	[35]
6D/7D	24.1	4D	7.6	79	Bread wheat	[12]

### Wheat pedigree and molecular phylogeny in this study

The ten cultivars from Kazakhstan were specifically chosen for this study for their productivity potential in drought-prone environment and reports of stable yields across multiple generations [60]. Eight popular cultivars were developed and produced relatively recently by breeders in Kazakhstan, while two much older, reference-pedigree cultivars were also selected.

Erythrospermum 841 (*ERY-841*) is one of the oldest reference-pedigree cultivars and may actually represent a landrace. Its original form was found as local germplasm in the Ashkhabad region (Turkmenistan) in 1913 and released as a cultivar in 1929 (Table 1). *ERY-841* has been characterised as being tolerant to drought and was used as a standard variety for many years during the Soviet Union era [61,62]. *ERY-841* also has potential salinity tolerance, although this has not been well documented. It was used as a pedigree-parent for at least 37 cultivars, with the last report of a registered cultivar in 1939, Lutescence 96 [63]. *ERY-841* has not been used directly for generating breeding lines since around 1978, due to the availability of many new cultivars with more advanced characteristics (P. Malchikov, Personal com.). It is possible that some cultivars used in this study were developed with breeding material derived from early crosses made using *ERY-841*, but pedigree analysis indicates that *ERY-841* is genetically distant from all of the other studied genotypes (Figure 4) which would imply selection against *ERY-841* alleles in recent years. Comparative analysis with a small panel of international wheats indicated that *ERY-841* is a member of the ‘historical-geographical’ clade A, containing older Asian wheat genotypes. The Central Asian country Turkmenistan (origin of initial germplasm of *ERY-841*) is geographically close to Uzbekistan, China, India and Pakistan, and therefore, their similarity is not unexpected. Sonora is one of the oldest wheat cultivars, with originating as far back as 1770 in Mexico prior to its transfer to the USA [47]. It is unclear why Sonora sits in the same clade A, together with *ERY-841* and other Asian genotypes. We can speculate that Asian genotypes in clade A with *ERY-841* had common ancestry with Sonora. The modern American cultivar Survivor (released in 1991) may help shed more light at this phylogenetic story but current record of the pedigree does not point to Sonora [47].

The second reference-pedigree cultivar was Saratovskaya 29 (*SAR-29*), which has a shorter history than *ERY-841* (released in 1957). This cultivar was extremely popular in the former USSR but it has been replaced by more advanced cultivars based on the same germplasm, such as Saratovskaya 60 (released in 1995 [46]). *SAR-29* was widely used for hybridisation and selection of new cultivars until relatively recently. *SAR-29* was included in a study of genetic polymorphism using SRAP markers, confirming the wide use of this germplasm for the production of new bread wheat cultivars in Russia [37]. In our study, *SAR-29* had specific patterns of unique SNP marker (Table 3) and SNP cluster distribution (Figure 1), not present in any of the other studied lines. This may indicate that some genetic fragments, particularly from the very distal parts of both arms of chromosome 2A, were never introgressed into modern wheats in Kazakhstan. We speculate that these relatively small genetic fragments may contain deleterious alleles or represent regions of important new alleles from other sources.

In our study, *SAR-29* clustered in the middle of sub-clade C2 much closer to the eight modern cultivars in the phylogenic tree than *ERY-841* (Figure 4). The sub-clade C2 was very distinct and contained cultivars from Kazakhstan and only a single Chinese accession (PI-447382). By contrast, the most closely related sub-clade C1 contained Australian and Chinese genotypes but none from Kazakhstan. This may indicate common genetic sources. *SAR-29* formed cluster C2-2 with AST-2. AST-2 was generated from three rounds of backcrossing with a progenitor-cultivar, Tzelinnaya yubileinaya, which has *SAR-29* in its parentage. *SAR-29* was also a direct parent of KAZ-19 (Table 1), although our analysis indicates that it is not as genetically related as AST-2. In fact, KAZ-19 fell into a separate sub-clade B2, along with genotypes from North America (USA and Canada). This intriguing observation may be explained by the presence of a common ancestor Kanred (USA, released in 1917) in the pedigrees of both KAZ-19 and the American cultivar Thatcher (released in 1934) [47]. However, KAZ-15 has the same ancestor but belongs to another sub-clade, C2. The results suggest that there was strong selection in Kazakh breeding programs for some genome regions found in North American germplasm leading to the observed position of KAZ-19 in the phylogeny. This could represent a fruitful avenue for further analysis and breeding.

Two clades (B1 and D) contained cultivars from North America (USA and Canada), and from Australia and the USA, respectively, demonstrating that studied genotypes from Kazakhstan were distributed in other clades. Interestingly, Chinese cultivars were relatively widely spread across clades A, C1 and C2 in our study (Figure 4).

Previous parentage analyses for Kazakhstan wheat genotypes were based either only on genealogical pedigree study [64], or on molecular-genetic analyses using 24 SSR markers [60] and 33 SSR markers [65], where eight of the wheat cultivars in these studies matched those included here. The different approaches showed some similarities. For example, in the present study, two cultivars *ERY-841* and KAZ-19 (clades A and B2, Figure 4) were most genetically distanced from the others, and these results were similar to those in the genealogical study [64] and to our previously published analysis using 24 SSR markers [60]. By contrast, three cultivars from sub-clade C2-4 in the current study (KAR-90, PAV-93 and TZE-3C) also combined as cluster III in the molecular-genetic analysis using 33 SSR markers [65], but they were dispersed into three groups (B, C and F) in the genealogical study [64]. The pedigrees for KAR-90, TZE-3C and PAV-93 give no indication of a shared genetic history but they did originate from close geographic locations in Kazakhstan. Therefore, it is possible that breeders sourcing similar parental material for their selections. Our observations of incomplete overlap between molecular and genealogical analyses are similar to a previous study using 20 elite wheat cultivars from wide geographic origin from Mexico (CIMMYT), India and China [66]. The authors reported relatively low but significant correlation between a genealogical pedigree study and molecular analysis using 93 SSR markers. There was greater consistency across the two studies for accessions that were more genetically distant than for those that were more closely related [66]. However, pedigree records are not always accurate, particularly for some older varieties, and many of the lines used in breeding programs carry quite high levels of heterozygocity or are mixtures of lines. The presented results of intra-varietal polymorphism using SNP marker technology confirm our previous findings [60]. Significant genetic polymorphism was identified in cultivar TZE-3C, while KAZ-R and KAZ-15 showed more moderate intra-cultivar polymorphism ([60] and current study). Intra-varietal polymorphism has important applications in breeding program for checking of genotype purity during development of commercial cultivars.

There is, therefore, likely to be some genetic drift over time and lines included in studies conducted today may have a very different genetic makeup when compared to the same line used many years ago in a breeding program.

### Unique SNP markers and distribution of their clusters

One aim of this study was to identify unique SNP markers specific for each cultivar; 885 were identified, 32.4% of the total number mapped. This is a slightly lower proportion but nevertheless comparable to other published proportions: 58.5% for 172 bread wheat accessions (303 unique SNPs of 518 mapped polymorphic SNPs [35]) and 56.1% for near-isogenic lines of durum wheat (43 unique SNPs of 82 polymorphic SNPs in total [36]). This is likely due to differences in the sets of SNP markers and germplasm used in the different studies.

The unique SNP markers and their clusters tended to be distributed on the distal regions of both arms for each chromosome rather than in centromeric regions, reflecting the distribution of recombination events. Our results are similar to those reported for the D genome of 225 accessions of *Aegilops tauschii* using 7,185 SNP markers [42], and consistent with the distribution of coding sequences in bread wheat [67].

To our knowledge, this is the first description of the distribution of unique SNP clusters to specific chromosomal regions within a set of germplasm (Figure 1). In our study, each of two cultivars, KAZ-R and PAV-93, had a single cluster on chromosomes 3BL and 3AS, respectively. Two clusters were found in different regions on each of chromosomes 4AS in TZE-3C and 2A in *SAR-29*. AST-2 had three clusters (chromosomes 4AS, 4BS and 5AS) while four clusters were found in each of KAZ-15 and KAR-90. The most genetically diverse cultivars were AKT-39, KAZ-19 and *ERY-841* with 11, 29 and 35 clusters of unique SNP markers, respectively. Identification of unique SNP markers and knowledge of the locations of unique clusters can be used as a first step in the preparation of cultivar-specific markers for bread wheat. The identification of similar unique SNP cluster regions in other populations may facilitate studies of the ancestry of such segments and indicate their significance in adaptation to specific environments.

## Conclusions

This SNP marker platforms provide a powerful tool for the molecular-genetic analysis of important agricultural crops such as bread wheat, due to the accuracy of the data and the relatively high-throughput. SNP markers can be employed for careful and precise identification of genetic phylogeny and differences between genotypes. The distribution of unique SNP markers and their clusters (regions containing more than three closely mapped SNPs) across chromosomes is an additional defining characteristic of particular genotypes. The current results, reporting SNP markers from ten cultivars of bread wheat from Kazakhstan, support the potential application of 9 K Infinium SNP marker technology for the study of larger panels of Kazakh wheat genotypes and for use in breeding programs in the country.

This initial analysis of current and historical wheat cultivars grown in Kazakhstan has confirmed the relative isolation of Kazakh germplasm from other wheat growing regions of the world. However, the study has also revealed some likely incursions of external germplasm notably from North America. The implication of these finding is that there may be considerable opportunity for genetic gain in Kazakh breeding program through systematic introgression and evaluation of elite lines from other programs. With a very large production area but currently relatively low yields, there is a significant opportunity to improve wheat production in Kazakhstan. It also appears that Kazakh breeders have made little use of global elite wheat germplasm and, therefore, there is an opportunity for significant genetic gain. The situation in Kazakhstan is likely to be reflected in a number of other regions where access to novel germplasm has been limited.

## Additional files

Additional file 1:
**List of selected bread wheat accessions from published data [** 3 **] for the phylogenetic comparison (Figure** **4** **).**
Additional file 2:
**Details of 9 K Infinium SNP array used in this study.**
Additional file 3:
**The data set supporting the results of this study.**

## Abbreviations

BAC: Bacterial artificial chromosome
DPI: Department of Primary Industries
EST: Expressed sequence tag
GBS: Genotyping-by-sequencing
KASPar: Competitive allele specific polymerase chain reaction
MAS: Marker-assisted selection, NGS, Next-generation sequencing
PIC: Polymorphism information content
SNP: Single nucleotide polymorphism
SSCP: Single strand confirmation polymorphism
SSR: Simple sequence repeats
SRAP: Sequence related amplified polymorphism
