# Supplementary material for: Application of next-generation sequencing technology to study genetic diversity and identify unique SNP markers in bread wheat from Kazakhstan
Source: BMC Plant Biol. 2014 Sep 28;14:258. doi: 10.1186/s12870-014-0258-7 (PMC4180858; doi:10.1186/s12870-014-0258-7)
Supplement: Additional file 1: — List of selected bread wheat accessions from published data [ 3 ] for the phylogenetic comparison (Figure 4 ). [file 12870_2014_258_MOESM1_ESM.doc]

**Additional file 1. List of selected bread wheat accessions from published data [3] for the phylogenetic comparison (Fig. 4)**

| **Country** | **Accession** | **Line** | **Status** | **Population / Growth habit** |
| --- | --- | --- | --- | --- |
|  |  |  |  |  |
| India | PI 322238 | G1112 | Cultivar | Asia / Spring |
| Pakistan | PI 520333 | G1103 | Cultivar | Asia / Spring |
| Uzbekistan | PI 24484 | G2575 | Landraces | Asia / Spring |
| Uzbekistan | PI 94530 | G1005 | Landraces | Asia / Spring |
|  |  |  |  |  |
| Australia | Annuello | G3129 | Cultivar | Australia / Spring |
| Australia | Axe | G3177 | Cultivar | Australia / Spring |
| Australia | Batavia | G1331 | Cultivar | Australia / Spring |
| Australia | Ega Stampede | G3126 | Cultivar | Australia / Spring |
| Australia | Hyden | G3158 | Cultivar | Australia / Spring |
| Australia | Krichauff | G3182 | Cultivar | Australia / Spring |
| Australia | Machete | G3128 | Cultivar | Australia / Spring |
| Australia | Sunvale | G3114 | Cultivar | Australia / Spring |
| Australia | Yitpi | G3519 | Cultivar | Australia / Spring |
|  |  |  |  |  |
| Canada | BW341 | G1812 | Cultivar | Canada / Spring |
| Canada | BW346 | G1813 | Cultivar | Canada / Spring |
| Canada | BW361 | G1814 | Cultivar | Canada / Spring |
| Canada | BW364 | G1815 | Cultivar | Canada / Spring |
| Canada | BW864 | G1807 | Cultivar | Canada / Spring |
| Canada | BW928 | G1810 | Cultivar | Canada / Spring |
| Canada | Canthatch | G1514 | Cultivar | Canada / Spring |
| Canada | Marquis | G2461 | Cultivar | Canada / Spring |
|  |  |  |  |  |
| China | PI 447382 | G1127 | Cultivar | China / Spring |
| China | PI 462147 | G1100 | Cultivar | China / Spring |
| China | PI 462148 | G1124 | Cultivar | China / Spring |
| China | PI 615321 | G1150 | Cultivar | China / Spring |
| China | PI 615323 | G1107 | Cultivar | China / Spring |
| China | PI 615327 | G1123 | Cultivar | China / Spring |
|  |  |  |  |  |
| USA | Amidon | G2596 | Cultivar | USA / Spring |
| USA | Calorwa | G2726 | Cultivar | USA / Spring |
| USA | Conan | G2594 | Cultivar | USA / Spring |
| USA | Ernest | G2587 | Cultivar | USA / Spring |
| USA | Lee | G2368 | Cultivar | USA / Spring |
| USA | Norpro | G2621 | Cultivar | USA / Spring |
| USA | Otis | G2710 | Cultivar | USA / Spring |
| USA | Sonora | G2469 | Cultivar | USA / Spring |
| USA | Survivor | G3030 | Cultivar | USA / Spring |
| USA | Thatcher | G2385 | Cultivar | USA / Spring |
